# The AO Spine TL Injury Classification System Drives Clinical Decision Making in Acute Thoracolumbar Fractures: A Retrospective Evaluation of Clinical Implementation

**DOI:** 10.1177/21925682261432541

**Published:** 2026-03-09

**Authors:** Vahid Buyukayten, Milan L. Ridderikhof, Jos W.R. Twisk, Olivier Q. Groot, Job N. Doornberg, Frank W. Bloemers

**Affiliations:** 1 Department of Emergency Medicine, 26066 Amsterdam UMC - Location AMC, Amsterdam, The Netherlands; 2 Department of Epidemiology and Data Science, 26066 Amsterdam UMC, Amsterdam, The Netherlands; 3 Department of Orthopaedic Surgery, Division of Surgical Specialties, 8124 University Medical Center Utrecht, Utrecht, The Netherlands; 4 Department of Orthopaedic Surgery, 10173 University Medical Center Groningen, Groningen, The Netherlands; 5 Department of Orthopaedic Trauma Surgery, 1065 Flinders University, Adelaide, Australia; 6 Department of Trauma Surgery, 26066 Amsterdam UMC - Location AMC, Amsterdam, The Netherlands

**Keywords:** AO spine thoracolumbar injury classification system, spine, acute thoracolumbar fractures, trauma, clinical decision-making, surgery, conservative treatment, adherence

## Abstract

**Study Design:**

Retrospective Cohort Study.

**Objectives:**

The primary objective was to examine the association between AO Spine Thoracolumbar Injury Classification System and treatment choice in patients with acute traumatic thoracolumbar fractures. The secondary objective was to evaluate the extent to which clinical decision-making adhered to the classification system’s recommendations.

**Methods:**

All adult patients who presented in the Emergency Department of a Level-I trauma centre with one or more acute thoracolumbar fractures between October 1st, 2016 and June 30th, 2021 were retrospectively included. Exclusion criteria were: pre-existing thoracolumbar fractures; missing data; patients who suffered non-survivable injuries; and pathological fractures. Potential confounders and effect modifiers included parameters related to patient and fracture characteristics. In addition, adherence in treatment decision-making to the classification system guideline was investigated.

**Results:**

A total of 553 patients were included of whom 30% were treated surgically. Overall, 68% of fractures were classified as A0-A3, 8.3% as A4, 6.2% as B1, 15% as B2, and 2.9% as C. Patients with A4 fractures underwent surgery more often than those with A0-A3 (OR:31.7; 95%CI 13.16-76.32; *P* < 0.01). None of the investigated variables were effect modifiers (all *P* > 0.10). Physicians’ adherence to the morphology components of the classification system varied between 86.4% and 100.0%.

**Conclusions:**

Patients with A4 fractures are treated surgically more frequently compared with those with A0-A3. While adherence to the morphological components of the classification system was excellent, documentation and application of the neurological and modifier components remained limited, underscoring the need for improved implementation in clinical practice in our hospital.

## Introduction

The global incidence of traumatic vertebral fractures was 10.5 per 100 000 inhabitants in 2018, corresponding to approximately 768 000 new cases per year.^1^ The most common localisation of a vertebral fracture is the thoracolumbar region (Th10-L2).^2^ Vertebral fractures are debilitating, including pain, deformity and have high mortality. Additionally, these fractures may have great impact on the psychological, economic, and social domains of both patients and their families.^3^

Traumatic thoracolumbar fractures can be treated either conservatively or surgically. In order to guide treatment decisions, various fracture classifications have been developed.^4^ The two most commonly used systems for categorizing these fractures and guiding treatment decisions are the ‘AO Spine Thoracolumbar Injury Classification System’ (AO Spine TL Classification System) and the ‘Thoracolumbar Injury Classification and Severity Score (TLICS).^5^ The AO Spine TL Classification System classifies fractures based on morphological (type A, B, or C), neurological (N0, N1, N2, N3, N4, or Nx) and (clinical) modifier (M1 or M2) components which are important in treatment decisions (Appendix Table 1).^6^ It is important to note that the scores of the classification systems are not designed to predict the choice of treatment but rather serve as guidelines offering treatment recommendations for either operative or conservative treatment.^7^

Despite international validation and widespread use of these classification systems, the optimal management of various thoracolumbar fractures remain a matter of debate. Specifically, the management of thoracolumbar burst fractures without neurological injury or disease modifiers (ie, AO Spine TL Classification System types A3N0M0 and A4N0M0) remains contentious.^8-10^ A meta-analysis concluded that 9% of patients with a thoracolumbar burst fracture without neurological injury who were initially treated conservatively, ultimately required surgery.^8^ In addition, an international survey of 483 spinal surgeons in 6 continents revealed regional differences in treatment recommendations for type A4N0M0 fractures. The results showed that spinal surgeons recommended surgery as treatment in 52% in South America, 46% in Europe, and none in North America.^6^ In addition to these regional differences, both patient and surgeon preferences play a role in treatment decision-making.^8,11,12^ A systematic review of Curfs et al concluded that the AO Spine TL Classification System, including patient-related modifiers, appears promising for use in clinical practice. However, the authors recommended further evaluation of its clinical usefulness and consideration of incorporating additional relevant parameters associated with worse outcome.^12^

Therefore, the primary objective of this study was to examine the association between the AO Spine TL Classification System and the choice of treatment — surgical vs conservative — in patients with acute traumatic thoracolumbar fractures. The secondary objective was to evaluate the extent to which clinical decision-making in this patient population adhered to the recommendations of the AO Spine TL Classification System.

## Methods

### Ethics and Guideline

As the current study was not a ‘medical research involving human acts (WMO)’ due to its retrospective design, the study did not require approval from the Institutional Review Board and a waiver was obtained (waiver no. W21_381#21.423). For this study the ‘Strengthening the Reporting of Observational studies in Epidemiology’ (STROBE) guideline was adhered to (Appendix Table 2).^13^

### Study Design and Patient Population

All adult patients (≥ 18 years) who presented in the Emergency Department of both locations of a Level-I trauma centre in the Netherlands between October 1st, 2016 and June 30th, 2021 with one or more acute (within 48 hours) thoracolumbar fractures (Th1-L5) were retrospectively included.^14^ Exclusion criteria were: (1) pre-existing thoracolumbar fractures, (2) missing data, (3) patients who suffered non-survivable injuries, (4) and pathological fractures due to spinal metastases.

### Outcomes

The outcome for the primary objective was the definitive choice of fracture treatment, classified as surgery or conservative treatment. Surgery was defined as any surgical intervention that involved direct manipulation and/or fixation of the fractured spine. Conservative treatment was defined as any treatment approach that did not involve surgical intervention to address the fracture such as bracing and/or physiotherapy. The choice of treatment was made through shared decision-making with the patient by the treating emergency physician or surgeon. The outcome for the secondary objective was the evaluation regarding treatment decision-making and adherence to the AO Spine TL Classification System.^15^

### AO Spine TL Classification System

The AO Spine TL Classification System distinguishes 3 categories of fractures, type A: compression injuries; type B: tension band injuries and type C: translational injuries (Appendix Table 1). The type A and B fractures are subcategorized into respectively 5 and 3 subtypes. This classification system provides a recommendation for surgical or conservative treatment. A cumulative score of ≤ 3 points recommend conservative treatment, while a score of ≥ 6 points recommend surgical intervention as the preferred initial treatment. Scores of 4 or 5 are “indeterminate”, with decision-making left to the discretion of the treating physician (Appendix Table 1).^15^

In this study, the total scores, as determined by at least three treating physicians (radiologist, emergency physician, trauma surgeon, orthopaedic surgeon, neurosurgeon or neurologist) and documented in the electronic health records were utilised. If this was not done or if there was a difference in these scores, two researchers (V.B. and another researcher) not involved in direct patient care recorded the scores independently. The most frequently occurring scores of the classification system were recorded in the database. This approach contributed positively to the inter-rater reliability.

### Parameters

Based on expert knowledge and relevant parameters, parameters - encompassing both patient and fracture characteristics - were selected as potential determinants of treatment decision-making.^16-19^ Patient characteristics included: sex, age, comorbidities, trauma mechanism, Glasgow Coma Score (GCS), and Injury Severity Score (ISS). Age was dichotomized into younger than 65 years and 65 years or older.^19^ Comorbidities were dichotomously categorised (yes or no), based on whether the patient had at least one of the following diseases: osteoporosis, diabetes mellitus, chronic obstructive pulmonary disease (COPD), asthma, rheumatoid arthritis, and ankylosing spondylitis.^16-18^ Trauma mechanism was categorised into fall less than 3 meters, fall more than 3 meters, traffic accident, and penetrating injury. GCS was categorised into 15 and 14 or less. ISS was categorised into 15 or less, and 16 or more. Fracture characteristics included: fracture level, number of fractures, fracture classification according to the AO Spine TL Classification System,^15^ presence of neurological injury and presence of multiple thoracolumbar fractures. Neurological injury is a component of AO Spine TL Classification System. In case of multiple fractures, the most severe type (regarding the AO Spine TL Classification System) and the corresponding fracture level were used in the analyses, as this determined treatment decisions. Information about patient and fracture characteristics were retrieved from imaging (Computed Tomography scan and/or X-ray) and medical history, and characteristics of physical examination were retrieved from the electronic health records as well.

### Statistical Analysis

**Figure 1.**
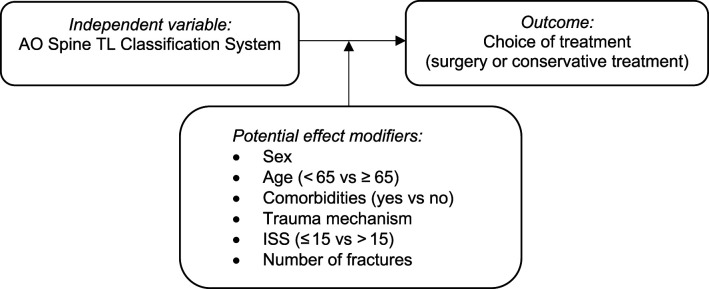
Overview of the primary study question with the association model between the independent variable AO Spine TL Classification System and the outcome choice of treatment, with the potential effect modifiers. Injury Severity Score (ISS)

Categorical data were presented as absolute numbers and percentages. The relationship between the independent variable AO Spine TL Classification System and the outcome choice of treatment (surgery vs conservative treatment) was analysed with logistic regression analysis. Both a crude and an analysis adjusted for age, sex, comorbidities, trauma mechanism, ISS, and number of fractures were performed. In addition, potential effect modification with the same variables was investigated (Figure 1).

Adherence to the AO Spine TL Classification System was calculated as the proportion of cases treated (conservatively or surgically) in line with its recommendations. Adherence was categorised using the following cut-off values: < 30% indicating poor adherence, 30-80% indicating intermediate adherence, and > 80% indicating excellent adherence.^20^

All patients meeting the inclusion and exclusion criteria were included to ensure as much available data as possible was collected.

In all analysis, *P*-value of ≤ 0.05 was used to determine statistical significance and a *P*-value ≤ 0.10 was used to determine significant effect modification. All statistical analyses were performed using SPSS IBM Version 28.0.

## Results

### Patient and Fracture Characteristics

**Figure 2.**
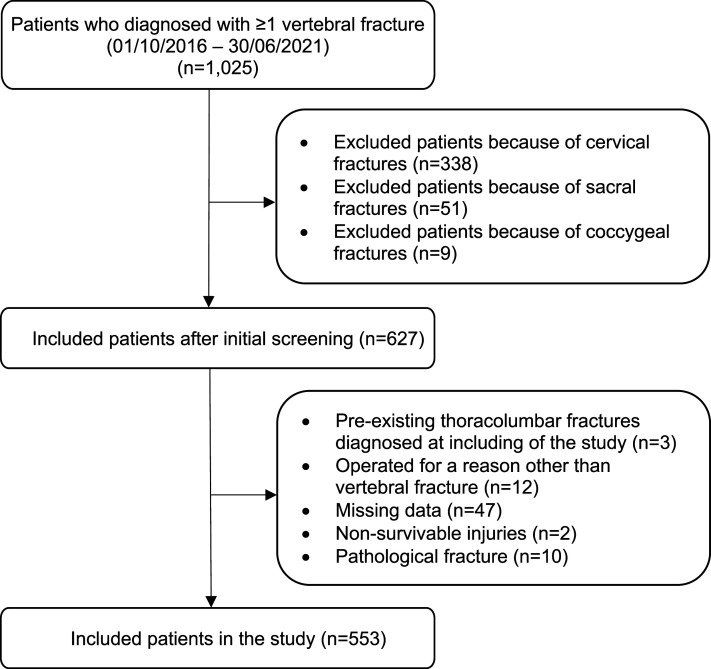
Overview of the study selection

In total, 553 patients were included of whom 58% were male and 345 (62%) were aged < 65 years (Figure 2; Table 1). Fractures were localised in the thoracic spine in 55% of the cases and in the lumbar spine in 45%. The vast majority of the patients had type A0, A1, A2, or A3 fractures. In the total study population, 30% of the patients were treated surgically.

**Table 1.** Baseline Characteristics Between the Conservative and Surgically Treated Group

Parameters	Conservative *n* = 387	Surgery *n* = 166	Total *n* = 553
Sex			
- Male	197 (51%)	121 (73%)	318 (58%)
- Female	190 (49%)	45 (27%)	235 (42%)
Age			
- < 65 years	219 (57%)	126 (76%)	345 (62%)
- ≥ 65 years	168 (43%)	40 (24%)	208 (38%)
Comorbidities			
- No	286 (74%)	139 (84%)	425 (77%)
- Yes	101 (26%)	27 (16%)	128 (23%)
Trauma mechanism			
- Fall < 3 meters	174 (45%)	34 (21%)	208 (38%)
- Fall ≥ 3 meters	64 (17%)	69 (42%)	133 (24%)
- Traffic accident	120 (31%)	59 (36%)	179 (32%)
ISS score			
- ISS ≤ 15	335 (87%)	115 (69%)	450 (81%)
- ISS ≥ 16	52 (13%)	51 (31%)	103 (19%)
GCS score			
- 15	344 (89%)	126 (76%)	470 (85%)
- < 15	43 (11%)	40 (24%)	83 (15%)
Fracture level			
- Thoracic	208 (54%)	94 (57%)	302 (55%)
- Lumbar	179 (46%)	72 (43%)	251 (45%)
Number of fractures			
- 1	159 (41%)	33 (20%)	192 (35%)
- 2	67 (17%)	25 (15%)	92 (16%)
- ≥ 3	161(42%)	108 (65%)	269 (49%)
Neurologic injury			
- No	387 (100%)	128 (77%)	515 (93%)
- Yes	0 (0%)	38 (23%)	38 (6.9%)
AO Spine TL classification System			
- A			
- A0, A1, A2, A3	356 (92%)	20 (12%)	376 (68%)
- A4	17 (4.4%)	29 (18%)	46 (8.3%)
- B			
- B1	3 (0.8%)	31 (19%)	34 (6.2%)
- B2, B3	11 (2.8%)	70 (42%)	81 (15%)
- C	0 (0%)	16 (9.6%)	16 (2.9%)

Injury Severity Score (ISS); Glasgow Coma scale (GCS)

### AO Spine TL Classification System

Due to the small number of patients in certain fracture types, and considering the similar treatment recommendations as defined by the AO Spine TL Classification System, the groups A0, A1, A2, and A3 classification system were combined. For the same reason, statistical analysis of B- and C-type of fractures, GCS, neurological injury, and penetrating injury as trauma mechanism was not possible.

Patients with AO Spine TL Classification System type A4 fractures were operated on more often than patients with type A0, A1, A2, or A3 fractures (adjusted OR 31.7; 95% CI 13.16-76.32; *P* < 0.01) (Table 2). No significant effect modification was found for all potential effect modifiers (all *P* > 0.10).

**Table 2.** AO Spine TL Classification System Type a With the Potential Effect Modifiers, Odds Ratio’s and 95% Confidence Interval

	Crude analysis			Adjusted analysis		
	*P*-value	OR	95% CI	*P*-value	OR	95% CI
Type A fracture (A4 vs A0, A1, A2 or A3)	< 0.001	30.37	14.36-64.23	< 0.001	31.69	13.16-76.32
Potential effect modifiers						
Age (≥ 65 vs < 65 years)	N/A	N/A	N/A	0.12	0.22	0.03-1.49
Sex (female vs male)	N/A	N/A	N/A	0.87	0.86	0.14-5.21
Comorbidities (yes vs no)	N/A	N/A	N/A	0.51	0.46	0.04-4.76
Trauma mechanism (fall ≥ 3m vs fall < 3m)	N/A	N/A	N/A	0.60	0.55	0.06-5.06
Trauma mechanism (traffic accident vs fall < 3m)	N/A	N/A	N/A	0.78	0.73	0.08-6.76
ISS (> 15 vs ≤ 15)	N/A	N/A	N/A	0.99	1.02	0.11-9.06
Number of fractures (2 vs 1)	N/A	N/A	N/A	0.50	2.64	0.16-43.17
Number of fractures (≥ 3 vs 1)	N/A	N/A	N/A	0.18	3.81	0.55-26.56

Odds ratio’s (OR); 95% confidence interval (CI); Injury Severity Score (ISS)

### Adherence to AO Spine TL Classification System

The adherence to the morphology components of AO Spine TL Classification System variated between 86.4% and 100.0% (Table 3). Despite guideline recommendations, 20 of the 376 (5.3%) patients with type A0-A3 fractures underwent surgical treatment, whereas 11 of the 81 (13.6%) patients with type B2-B3 fractures received conservative treatment.

**Table 3.** Adherence to AO Spine TL Classification System to the Morphological Components

Types of fractures in AO spine TL classification system	Treatment recommendation (conservatively and/or surgically) based on morphology components of the classification system	Conservatively treated group (*n* = 387)	Surgically treated group (*n* = 166)	Adherence to the treatment recommendation based on the morphological components of the classification system
A				
A0, A1, A2 A3	Conservative	356	20	95%
A4	Both^a^	17	29	Not applicable
B				
B1	Both^a^	3	31	Not applicable
B2, B3	Surgically	11	70	86%
C	Surgically	0	16	100%

^a^Definitive choice of treatment is left to the discretion of the physician.

## Discussion

Although the AO Spine TL Classification System is an internationally validated treatment algorithm, the treatment of types A3N0M0 and A4N0M0 remains controversial.^8^ In our clinical practice we recognise the uncertainties of the AO Spine TL Classification System in relation to A4N0M0 fractures, as the definitive treatment is left to the discretion of the treating physician.^8-10^ First, this study identified that patients with type A4 fractures were more frequently treated surgically compared to patients with A0, A1, A2, or A3 fractures. This was to be expected to some extent. Second, in clinical practice adherence to the neurological components, the modifier components and the overall scoring algorithm of the AO Spine TL Classification System is still unknown, whereas adherence to the morphological components of the classification system appears excellent.

To address the primary objective, the findings of our study are reasonably in line with the findings of Kweh et al.^10^ The differences concern the determination of clusters of classifications from the AO Spine TL Classification System that are used within the analyses. In the study of Kweh et al, the AO Spine TL Classification System types A3 and A4 fractures were grouped together and compared to A0, A1 and A2 fractures. They found that types A3 and A4 fractures are more frequently treated surgically than A0, A1 and A2 fractures. In our study, due to the sample size of each type, we grouped A0, A1, A2, and A3 fractures together and compared them with A4 fractures. In our opinion, this grouping was justified by the AO Spine TL Classification System treatment recommendations, which are the same for A0, A1, A2, and A3 fractures, provided that the patient is neurologically intact and no modifiers are present. Despite the differences in determination of clusters of classifications, we believe our findings are in essence in line with those reported by Kweh et al.^10^ We found that type A4 fractures are more frequently treated surgically than the A0, A1, A2, and A3 fractures. The reason for this is that it was observed that all patients with A4 fractures who underwent surgery had neurological injury identified in physical examination and/or fragment dislocation into the spinal canal detected in imaging. The above-mentioned finding may suggest that the neurological component of the AO Spine TL Classification System is an essential component in treatment decision-making for A4 fractures. The study of Kweh et al suggests that A4 fractures are more frequently treated surgically due to the fact that A3 fractures are more equivalent to B fractures, compared to A3 fractures.^10^ The findings of our study support that explanation. Our study however differs from that of Kweh et al on the source of data. They used a questionnaire to collect their data,^10^ our data originated from electronic health records.

To address the secondary objective, the current study found that in treatment decision-making the adherence to the morphology component of the AO Spine TL Classification System appears excellent, with compliance exceeding 80%. For the A4 and B1 fractures the adherence to the morphology component could not be determined, as the definitive treatment choice is left to the discretion of the treating physician. However, the adherence to the neurological components, the modifier components and the overall scoring algorithm of the AO Spine TL Classification System cannot be determined due to a lack of documentation in our electronic health records. Consequently, it was due to the retrospective design of the study not possible to determine whether these components and the overall scoring algorithm were applied by treating physicians in clinical practice but not documented, or that they were not adhered to at all.

In cases where the treatment decision deviated with the treatment recommendation of AO Spine TL Classification System classification A, it was observed that patients had a neurological injury and/or an unstable fracture, the underlying cause of which was not documented. All patients with AO Spine TL Classification System types B fracture (except those in B1) and C with neurological injury were treated operatively, which is consistent with the treatment recommendation of AO Spine TL Classification System for these types of fractures. Although the influence of neurological injury on treatment decision could not be statistically analysed due to a relatively small number of patients, the results of visually inspecting the data suggests neurological injury is a crucial factor in treatment decision-making. This finding has also been found, as earlier mentioned, in the treatment decision-making of A4 fractures. In cases where the treatment decision deviated with the treatment recommendation of AO Spine TL Classification System classification B, it was observed that most patients aged ≥ 75 years and presented with multiple comorbidities, such as cardiac pathology or poor bone quality. Moreover, in one case, the presence of a hematoma for wound healing and an increased International Normalized Ratio (INR) value has led to a conservative treatment instead of surgery, which was the treatment recommendation of the AO Spine TL Classification System.

The selection of the variables is based on literature and on the components of the AO Spine TL Classification System. For example, COPD and diabetes mellitus are risk factors for developing pulmonary complications after spine surgery.^16^ Corticosteroids are commonly used medications for asthma and COPD and are associated with developing osteoporosis and series of vertebral fractures.^17^ Additionally, osteoporotic fractures are associated with increased morbidity and mortality.^18^ Ankylosing spondylitis is a modifier component in the AO Spine TL Classification System. Male sex, high-energy trauma mechanism, lower GCS and higher ISS are associated with an increased 1-year mortality in patients with thoracolumbar fractures.^21^ Multiple thoracolumbar fractures are associated with adverse events after a thoracolumbar surgery as treatment.^22^

This study had several limitations of which the most important was the retrospective study design and its inherent bias regarding documentation in electronic health records. Neurological and modifier components, important in treatment decision-making according to the classification system were not always documented. Second, this study was conducted in a single Level-I trauma centre, that could have led to selection bias and might restrict the generalizability of the study findings. Third, although the intention was to analyse the AO Spine TL Classification System Types B and C, this was not deemed possible due to a relatively small number of patients in these categories. Therefore, it remains unclear whether fracture types B and C have confounding and/or effect modification in choice of treatment. In addition, we initially intended to analyse neurological injury and GCS as a potential confounder and/or effect modifiers in the analyses. However, as previously stated this was not possible due to a relatively small number of patients. As a result, it remains unclear whether neurological injury and GCS are an effect modifier and/or confounder in these analyses. In addition, we initially also intended to analyse penetrating injury as a trauma mechanism. However, this was not possible due to a relatively small number of patients. Fourth, in order to correct for small patient groups, A0-A3 types of fractures were grouped together. However, dichotomising these categories may have led to loss of information, and study findings might have been influenced. Finally, regarding the selected comorbidities (osteoporosis, asthma, COPD, diabetes mellitus, rheumatoid arthritis, and ankylosing spondylitis), these were analysed collectively, which limits the ability to assess the specific impact of different combinations. As a result, the effects of multiple coexisting comorbidities remain unclear.

Despite these limitations, this study emphasizes the importance of using AO Spine TL Classification System in clinical practice, in guiding treatment decisions for patients with acute traumatic thoracolumbar fractures. Furthermore, to our knowledge this is the first large cohort study investigating confounding and effect modification in the relation between AO Spine TL Classification System and definitive fracture treatment in patients with acute traumatic vertebral fractures. This study recommends comprehensive documentation of the neurological and modifier components of the AO Spine TL Classification System in our hospital and recommend further study on this field. Future prospective multi-centre studies, particularly in international clinical settings, may provide treatment recommendations on particularly A3N0M0 and A4N0M0 fractures.

## Conclusion

Patients with AO Spine TL Classification System type A4 fractures, based on the morphology components of the classification system, were treated surgically more frequently than type A0, A1, A2, or A3 fractures. The analyses of patient and vertebral fracture characteristics showed no effect modifiers. Most classic fracture classification systems do not drive surgical decision-making in clinical practice, making them primarily useful for inter-surgeon communication and research documentation. The AO Spine TL Classification System does drive treatment.

While adherence to the morphological components of the AO Spine TL Classification System was excellent, explicit documentation of the neurological and modifier components remained suboptimal. This underscores the need for improved documentation of the use of the AO Spine TL Classification System in clinical practice in our hospital, which would enable our hospital to conduct a future study to evaluate the implementation of the neurological components, the modifier components and the overall scoring algorithm of the AO Spine TL Classification System.

## Supplemental Material

Supplemental Material - The AO Spine TL Injury Classification System Drives Clinical Decision Making in Acute Thoracolumbar Fractures: A Retrospective Evaluation of Clinical Implementation Supplemental Material for The AO Spine TL Injury Classification System Drives Clinical Decision Making in Acute Thoracolumbar Fractures: A Retrospective Evaluation of Clinical Implementation by Vahid Buyukayten, Milan L. Ridderikhof, Jos W.R. Twisk, Olivier Quinten Groot, Job N. Doornberg, Frank W. Bloemers in Global Spine Journal

Supplemental Material - The AO Spine TL Injury Classification System Drives Clinical Decision Making in Acute Thoracolumbar Fractures: A Retrospective Evaluation of Clinical Implementation Supplemental Material for The AO Spine TL Injury Classification System Drives Clinical Decision Making in Acute Thoracolumbar Fractures: A Retrospective Evaluation of Clinical Implementation by Vahid Buyukayten, Milan L. Ridderikhof, Jos W.R. Twisk, Olivier Quinten Groot, Job N. Doornberg, Frank W. Bloemers in Global Spine Journal

## Data Availability

Data can be made available on reasonable request.*
