# Supplementary material for: The AO Spine TL Injury Classification System Drives Clinical Decision Making in Acute Thoracolumbar Fractures: A Retrospective Evaluation of Clinical Implementation
Source: Global Spine J. 2026 Mar 9:21925682261432541. Online ahead of print. doi: 10.1177/21925682261432541 (PMC12971509; doi:10.1177/21925682261432541)
Supplement: Supplemental Material - The AO Spine TL Injury Classification System Drives Clinical Decision Making in Acute Thoracolumbar Fractures: A Retrospective Evaluation of Clinical Implementation [file sj-pdf-1-gsj-10.1177_21925682261432541.pdf]

Appendix

Table 1. Overview of the AO Spine Thoracolumbar Injury Classification System (AO-TLICS), original developed by Vaccaro et al. and updated by Morrissey et al.

Morphology	Points
Type A: Compression fractures	
• A0: Minimal injuries such as transverse process fractures	0
• A1: Wedge compression	1
• A2: Pincer compression injury	2
• A3: Incomplete burst fracture: fracture that only involves a single endplate	3
• A4: Complete burst fracture: fracture that involves both endplates	5
Type B: Tension band injuries	
• B1: Osseous disruption of the tension band	5
• B2: Posterior tension band injury including ligamentous injury	6
• B3: Anterior tension band injury	7
Type C: Translation injuries	8
Neurology	
• N0: Neurologically intact patients	0
• N1: Resolved transient neurological symptoms	1
• N2: Persistent radicular symptoms	2
• N3: Incomplete spinal cord injury or cauda equina injury	4
• N4: Complete spinal cord injury	4
• Nx: Neurologic exam is unobtainable	3
Modifiers	
• M1: covers compression type injuries in which the status of the posterior ligamentous complex is not clear	1
• M2: covers morbidities that can affect in patients the treatment algorithm, such as polytrauma	0
A summative score of 3 or less recommends nonoperative treatment while a score of 6 or more recommends operative intervention. A score of 4 or 5 is “indeterminate” with decision-making left to the discretion of the treating surgeon.	
